# Sages & Scholars: Connecting OLLI Members and Academic Students for Intergenerational Exchange

**DOI:** 10.1093/geroni/igaf122.878

**Published:** 2025-12-31

**Authors:** Tina Newsham, Amy Keith, Jennifer Heber-Brown

**Affiliations:** The University of North Carolina at Wilmington, Wilmington, North Carolina, United States; Osher Lifelong Learning Institute, Wilmington, North Carolina, United States; Osher Lifelong Learning Institute, Wilmington, North Carolina, United States

## Abstract

While the population is aging, higher education classrooms remain filled primarily with “traditionally-aged” students, which limits opportunities for exchange and shared learning experiences across generations. This limitation is detrimental to all students (and those who do not feel welcome or able to engage in higher ed classes) and is particularly unfortunate in gerontology classes where many students have limited connection with older adults outside their families. To foster intergenerational connection and exchange, gerontology students at the University of North Carolina Wilmington (UNCW) are partnered with members of the UNCW Osher Lifelong Learning Institute and asked to engage in a series of social activities in a program called Sages & Scholars. In this presentation, we share the history of the development of the Sages & Scholars program at UNCW and lessons learned through the first two years of implementation. We believe this program serves as a model for others seeking to implement innovative intergenerational initiatives on their campuses. Programs like Sages & Scholars foster meaningful connections across generations and contribute to age-inclusivity on college campuses.

